# Comparisons between dual-energy X-ray absorptiometry and bioimpedance devices for appendicular lean mass and muscle quality in Hispanic adults

**DOI:** 10.1017/S000711452400076X

**Published:** 2024-04-15

**Authors:** Bassel Nassar, Grant M. Tinsley, Kyung-Shin Park, Stefan A. Czerwinski, Brett S. Nickerson

**Affiliations:** 1 School of Health and Rehabilitation Sciences, The Ohio State University, Columbus, OH 43210, USA; 2 Department of Kinesiology and Sport Management, Texas Tech University, Lubbock, TX, USA; 3 College of Nursing and Health Sciences, Texas A&M International University, Laredo, TX, USA

**Keywords:** Skeletal mass, Muscular strength, Handgrip strength, Body composition, Muscular fitness

## Abstract

The purpose of this study was to compare single- and multi-frequency bioimpedance (BIA) devices against dual-energy X-ray absorptiometry (DXA) for appendicular lean mass (ALM) and muscle quality index (MQI) metrics in Hispanic adults. One hundred thirty-one Hispanic adults (18–55 years) participated in this study. ALM was measured with single-frequency bioimpedance analysis (SFBIA), multi-frequency bioimpedance analysis (MFBIA) and DXA. ALM_TOTAL_ (left arm + right arm + left leg + right leg) and ALM_ARMS_ (left arm + right arm) were computed for all three devices. Handgrip strength (HGS) was measured using a dynamometer. The average HGS was used for all MQI models (highest left hand + highest right hand)/2. MQI_ARMS_ was defined as the ratio between HGS and ALM_ARMS_. MQI_TOTAL_ was established as the ratio between HGS and ALM_TOTAL_. SFBIA and MFBIA had strong correlations with DXA for all ALM and MQI metrics (Lin’s concordance correlation coefficient values ranged from 0·86 (MQI_MFBIA-ARMS_) to 0·97 (Arms LM_SFBIA_); all *P* < 0·001). Equivalence testing varied between methods (e.g. SFBIA *v*. DXA) when examining the different metrics (i.e. ALM_TOTAL_, ALM_ARMS_, MQI_TOTAL_ and MQI_ARMS_). MQI_ARMS_ was the only metric that did not differ from the line of identity and had no proportional bias when comparing all the devices against each other. The current study findings demonstrate good overall agreement between SFBIA, MFBIA and DXA for ALM_TOTAL_ and ALM_ARMS_ in a Hispanic population. However, SFBIA and MFBIA have better agreement with DXA when used to compute MQI_ARMS_ than MQI_TOTAL_.

Muscular strength and appendicular lean mass (ALM) are often used to diagnose sarcopenia and calculate muscle quality^(1,2)^. The decline of muscular strength and ALM in ageing has resulted in most research being centred on older adults. In addition, poor muscle quality is associated with chronic diseases such as type II diabetes, osteoporosis and cardiovascular disease, all of which can have a profound impact on quality of life and activities of daily living^(3–5)^. These health conditions have led to an interest in measuring muscle quality in older populations. Nonetheless, young and middle-aged adults may also benefit from monitoring muscle quality, especially when seeking to improve functional capacity^(6)^. For instance, young adults have the greatest increase in the risk of chronic diseases^(7)^. Therefore, improving functional capacity is also an important preventative tactic for young-to-middle-aged adults. In addition, early identification of individuals with comprised strength and muscle functionality may help to reduce cost in public health services^(8)^. Collectively, these findings demonstrate the benefit of measuring muscle quality across various age spectra.

Methodological considerations are important to consider when assessing muscle quality. Further, the use of different methods, particularly body composition techniques, may yield different values when seeking to quantify muscle quality. For instance, muscle quality index (MQI), characterised by the ratio of muscular strength relative to skeletal muscle tissue, is often determined using dual-energy X-ray absorptiometry (DXA) for the latter component^(6,9,10)^. Nonetheless, alternative approaches for body composition, such as bioimpedance analysis (BIA), can be used as an alternative to DXA for computing MQI^(11,12)^. The utilisation of different body composition methods across studies can make comparisons of previous findings challenging. For example, conflicting MQI results between studies could be attributed to the utilisation of different body composition methods, instead of differences in characteristics between study cohorts.

Numerous studies have compared BIA and DXA for total and regional body composition metrics such as body fat, lean mass and bone mineral content^(13–19)^. For example, research has shown the accuracy of single-frequency bioimpedance analysis (SFBIA) for predicting appendicular lean and fat mass varies based on sex and segmental mass^(15)^. In addition, researchers have shown that BIA is more accurate when utilised to predict lean mass instead of fat mass^(15,19,20)^. Lastly, validation research has shown BIA can be used to estimate bone mineral content, when compared with DXA, in healthy populations^(16,17)^. It is important to highlight that many validation studies on BIA have been completed in non-Hispanic populations. This could be problematic when seeking to generalise BIA devices in Hispanic adults who have differing fat-free mass characteristics than assumed constants (i.e. hydration = 73·8 % of fat-free mass), which are used to predict body composition via bioimpedance technology^(21)^. For instance, previous research has shown the hydration of fat-free mass varies from 63·76 to 79·55 % in Hispanic adults^(22)^. This could potentially have an impact on predicting body composition with BIA devices. Indeed, Nickerson and Snarr^(13)^ revealed multi-frequency bioimpedance analysis (MFBIA) has large proportional bias when estimating whole-body fat mass in Hispanic females. Despite these findings, the utilisation of BIA in Hispanic adults needs further exploration.

One area that has yet to be evaluated in Hispanic adults is the agreement between various MQI models when using DXA- and BIA-derived ALM. Determining whether simpler techniques such as BIA can be used as an alternative to DXA for MQI models could be very helpful in clinical settings that do not have access to the latter method. For example, the cost and maintenance of a DXA machine can be very expensive. In addition, DXA emits radiation, which may be contraindicated in certain clinical populations and requires certified/licensed operator in some jurisdictions. Consequently, the utilisation of DXA-derived ALM for determining MQI is limited to sophisticated clinical and research settings, which limits its application. As a result, more affordable, user-friendly and non-radiological body composition techniques such as BIA are increasingly popular for computing MQI. Accordingly, the purpose of this study was to compare single- and MFBIA devices against DXA for ALM and MQI metrics in Hispanic adults.

## Methods

### Participants

One hundred and thirty-one participants (71 F, 60 M) were included in the present analysis (Table 1). Eligible participants were (1) 18 – 65 years of age; (2) reported no cardiac, pulmonary, or metabolic diseases; (3) weight and height < 159 kg and 193 cm, respectively, due to DXA table restrictions; and (4) Hispanic descent. Recruitment occurred via flyers, word of mouth and classroom recruitment. All eligible participants in the present study successfully completed testing. Exclusion criteria included persons with non-disease-related conditions that may affect body composition, intra- and extra-cellular fluid or DXA measurements (i.e. those currently or recently pregnant, persons with limb amputations and individuals with implanted metallic devices). All participants provided written informed consent and completed a medical history questionnaire prior to participation in the study. This study was conducted according to the guidelines presented in the Declaration of Helsinki, and all procedures involving human subjects/patients were approved by the Institutional Review Board of the host university (IRB# 2021–03-16).

### Procedures

All research participants reported to the laboratory for data collection following pre-testing guidelines, which included (1) no high-intensity exercise for 24 h, (2) fasting ≥ 8 h, (3) no alcohol or caffeine for ≥ 24 h, (4) no water intake ≥ 2 h. The adherence to pre-testing guidelines for each participant was assessed via a questionnaire upon arrival at the laboratory. Once pre-testing guideline adherence was ensured, hydration (i.e. urine-specific gravity), anthropometric (i.e. height and body mass), SFBIA, MFBIA, DXA and muscular strength (i.e. handgrip strength (HGS)) assessments were completed. Prior to all anthropometric and body composition measurements, shoes, jewellery and metallic objects were removed to minimise measurement error. Hydration was assessed via urine-specific gravity using a hand-held refractometer (Atago SUR-NE, Atago Corp Ltd., Tokyo, Japan). Participants’ urine-specific gravity values had to fall within the range of > 1·004 and < 1·029 to complete testing^(23)^. Standing height was measured to the nearest 0·1 cm using a stadiometer (SECA 213, Seca Ltd., Hamburg, Germany).

### Multifrequency bioimpedance analysis

MFBIA was used to measure body mass (BM) to the nearest 0·1 kg. Moreover, MFBIA was the first body composition test completed. ALM_TOTAL_ (left arm + right arm + left leg + right leg) and ALM_ARMS_ (left arm + right arm) were computed based upon manufacturer’s instructions (InBody 570, InBody USA, Cerritos, CA). The MFBIA device employed in the current study utilised a tetrapolar 8-point tactile electrode system, which sends three frequencies (i.e. 5, 50, and 500 kHz) of alternating currents through the body. For testing, subjects’ feet were centred on the electrodes and the hand electrodes were grasped with arms being held wide enough so there was no contact between the arms and torso. The position was held for the duration of the test (approximately 45 s). Once the assessment was completed, participants were prompted to return the hand electrodes and step off the device.

### Dual-energy X-ray absorptiometry

Immediately after MFBIA testing, participants had their criterion ALM_TOTAL_ and ALM_ARMS_ derived using DXA (GE Lunar Prodigy; Software version 14.10.022; GE Lunar Corporation, Madison, WI, USA). Prior to each use, the DXA was calibrated according to manufacturer guidelines using a standardised calibration block. Participants were positioned supine on the DXA platform with arms resting along the sides of the body and feet secured with Velcro straps around the ankles to reduce movement for the duration of the scan. Reflection scanning was completed on any participant exceeding the scanning area of the DXA table. The positioning of participants receiving a reflection scan aimed to limit the amount of left side of the body (e.g. left arm) outside the scanning area of the DXA machine. After each scan, a trained technician manually adjusted regions of interest.

### Single-frequency bioimpedance analysis

After DXA scans, participants had ALM_TOTAL_ (left arm + right arm + left leg + right leg) and ALM_ARMS_ (left arm + right arm) measured with SFBIA (Quantum V, RJL systems, Clinton MI) while lying on the DXA table. For SFBIA testing, the participants’ right and left shoe and sock remained off, and their arms were placed ≥ 30° away from the body with legs separated and not touching. Excess hair at electrode sites was removed, and the skin was cleaned with alcohol pads and dried prior to electrode placement. Surface electrodes were placed on the right and left wrist beside the ulnar head and on the first joint of the middle finger. Surface electrodes were also placed on the right and left foot beside the medial malleolus and on the base of the second toe. Next, leads were attached to the eight electrodes and a single-frequency (i.e. 50 kHz) whole-body impedance measurement was obtained for each subject. ALM_TOTAL_ and ALM_ARMS_ were computed using the built-in SFBIA algorithm.

### Handgrip strength

All handgrip tests were completed using a hydraulic hand dynamometer (Jamar, Performance Health Supply Inc., Cedarburg, WI). Prior to each test, the dynamometer was adjusted so the second third, fourth and fifth digit of the hand (i.e. proximal interphalangeal joint) was bent 90°. To complete each test, participants were instructed to be in a standing position, hold the dynamometer with the elbow flexed at 90° and squeeze the dynamometer as hard as possible while avoiding the Valsalva manoeuvre^(24)^. HGS was recorded in kg and the dynamometer was reset to zero prior to the next test. This procedure was repeated with the opposite hand and repeated two additional times. The highest value of the three readings for each hand was averaged to compute HGS.


HGS=(highestlefthand+highestrighthand)/2

### Muscle quality index.

MQI_ARMS_ was defined as the ratio between HGS and ALM_ARMS_ (HGS/ALM_ARMS_) for each body composition device (i.e. SFBIA, MFBIA and DXA). MQI_TOTAL_ was established as the ratio between HGS and ALM_TOTAL_ (HGS/ALM_TOTAL_) for each body composition device (i.e. SFBIA, MFBIA and DXA).

### Statistical analysis

The linear relationships between DXA, MFBIA and SFBIA for all ALM and MQI variables were established using Deming regression, which accounts for errors in the measurement of both variables^(25)^, and compared with a perfect relationship (i.e. the line of identity). Pearson’s R^2^, RMSE and CCC values were also calculated. Equivalence testing^(26)^ was performed using TOST to determine if DXA, MFBIA and SFBIA variables were equivalent based on equivalence regions of 2·5 %, consistent with previous research^(27)^. Additionally, Bland–Altman analyses were performed,^(28)^ including estimation of the 95 % limits of agreement and linear regression to examine proportional bias. Associations between alternate MQI metrics were examined using Pearson’s correlations. Statistical analyses were conducted in R (version 4.3.1) using the *DescTools*,^(29)^ *deming*,^(25)^ and *TOSTER*^(26)^ packages. Values are presented as mean ± SD and statistical significance was accepted at *P* < 0·05.

## Results

### Total appendicular lean mass outcomes

**Fig. 1.**
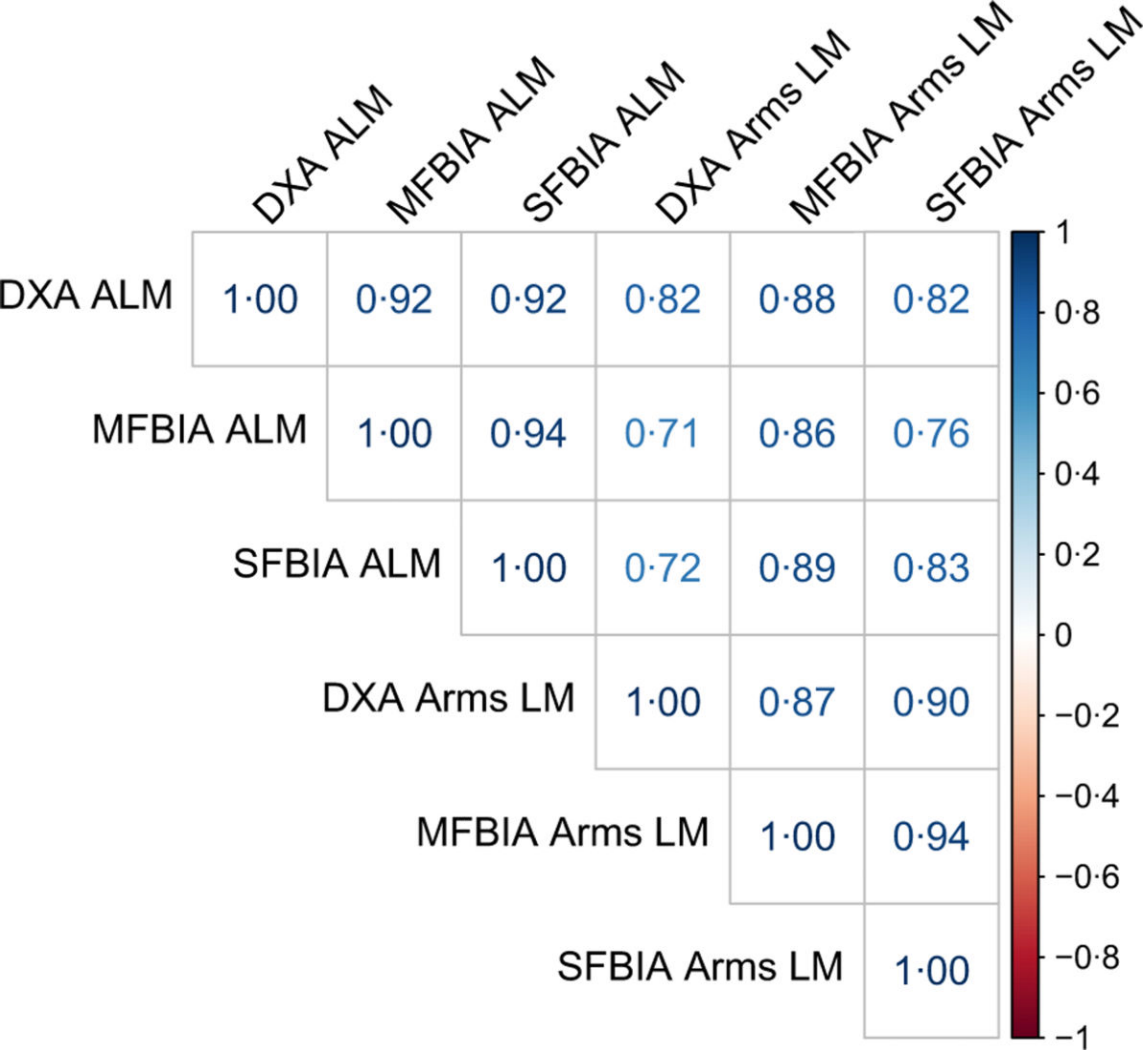
Correlation matrix. Correlations between dual-energy X-ray absorptiometry (DXA), single-frequency bioimpedance analysis (SFBIA) and multiple-frequency bioimpedance analysis (MFBIA) when measuring appendicular lean mass (ALM) and arms lean mass (LM).

**Fig. 2.**
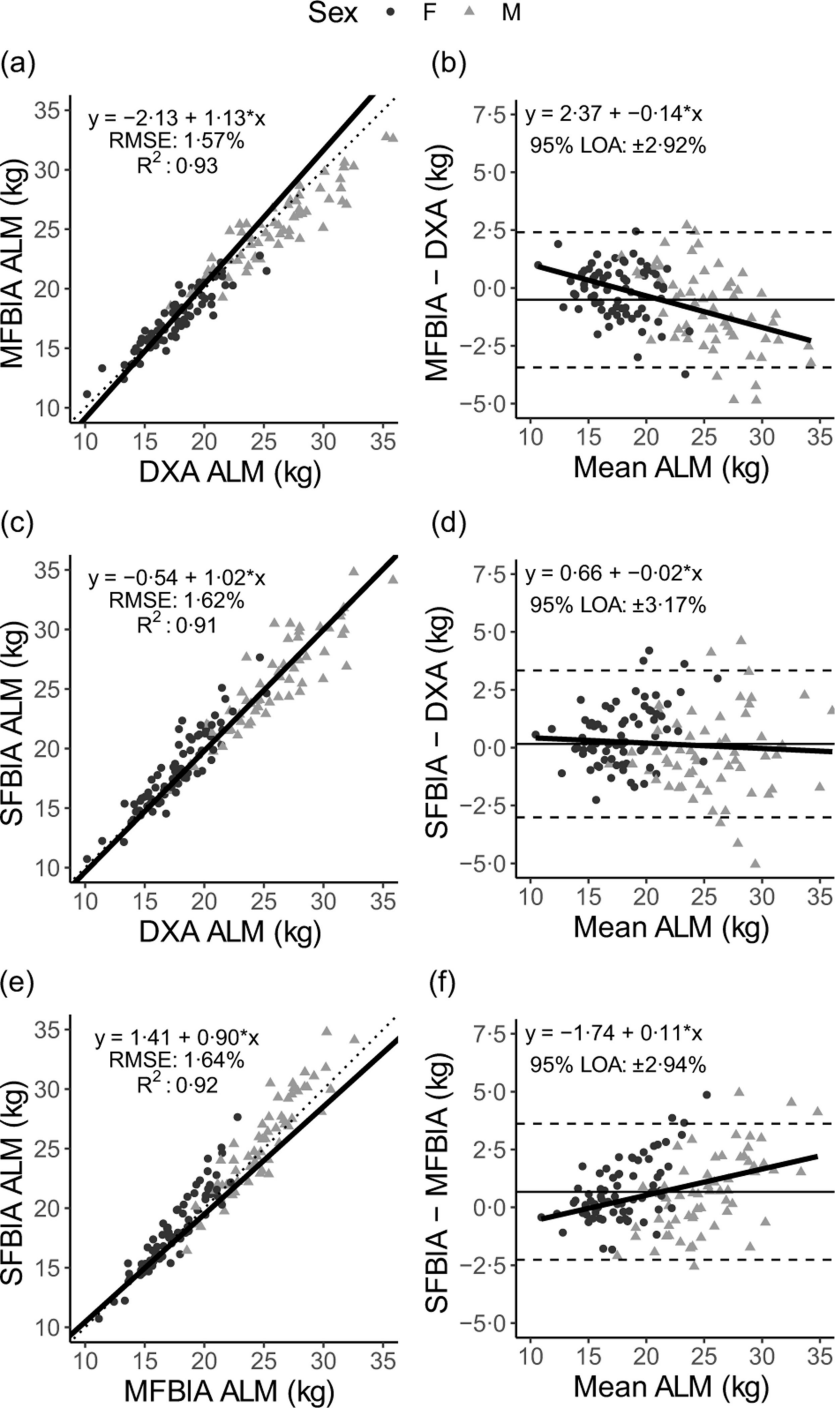
Comparison of body composition devices for estimating appendicular lean mass. *Line of Identity:* The ordinary least squares regression line as compared with the line of identity is displayed for single-frequency bioimpedance analysis (SFBIA), multi-frequency bioimpedance analysis (MFBIA) and dual-energy X-ray absorptiometry (DXA) comparisons. Root mean square error (RMSE) and coefficient of determination (*R*^2^) are also presented. Results of appendicular lean mass (ALM) are displayed for MFBIA *v*. DXA (a), SFBIA *v*. DXA (c), and SFBIA *v*. MFBIA (e). *Bland*–*Altman Analysis:* The relationship between the average of the ALM estimates and a reference method (*x*-axis) and the difference in the estimate minus that of the reference method (*y*-axis) is displayed. The linear regression line indicates the degree of proportional bias. Horizontal dashed lines indicate the upper and lower limits of agreement (LOA), and the horizontal solid line indicates the constant error between methods. Linear regression equations and 95 % LOA values are also displayed. Results of ALM are displayed for MFBIA *v*. DXA (b), SFBIA *v*. DXA (d) and SFBIA *v*. MFBIA (f).

**Fig. 3.**
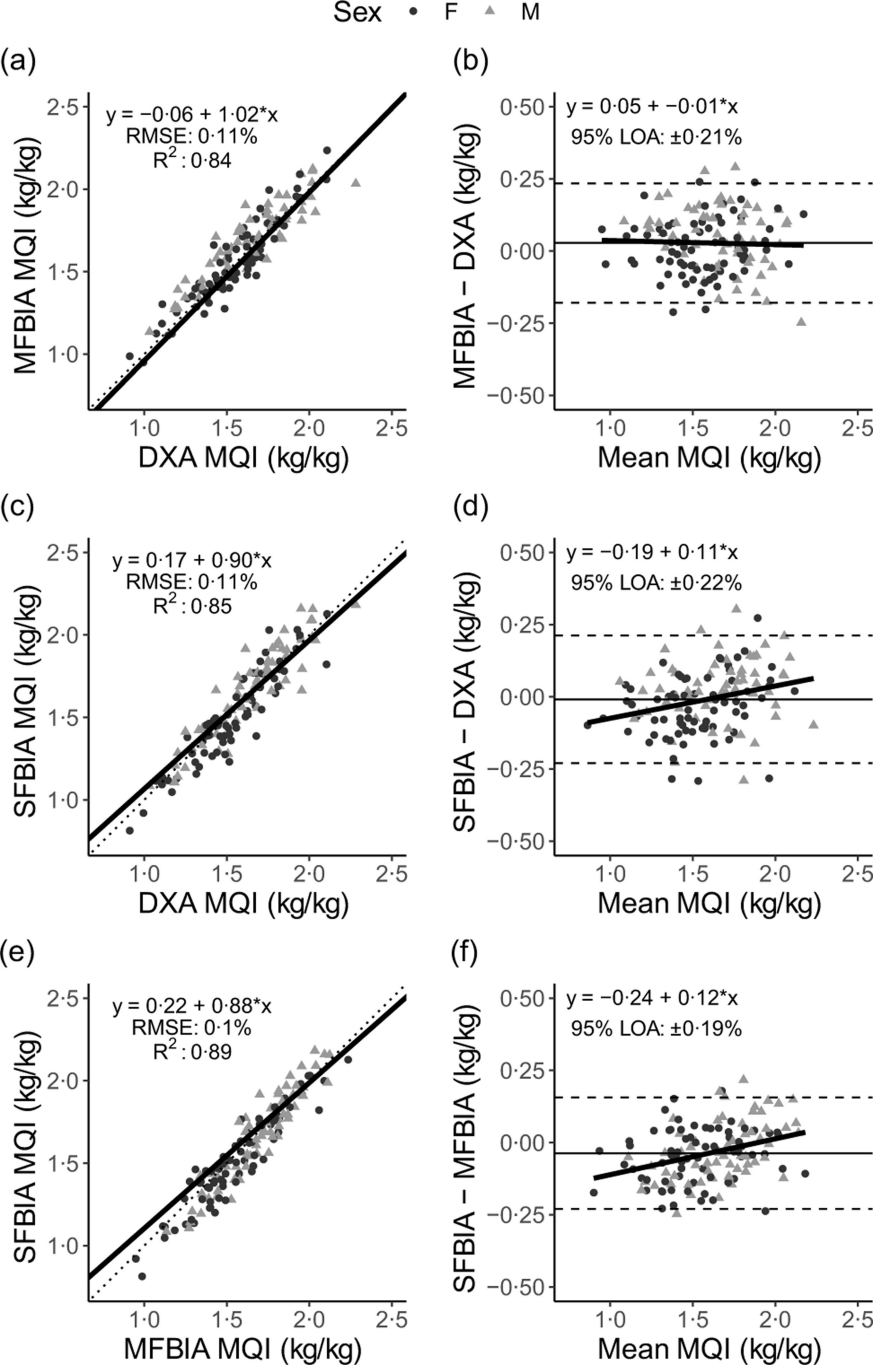
Comparison of body composition devices for measuring muscle quality index in arms and legs (MQI_TOTAL_). *Line of Identity:* The ordinary least squares regression line as compared with the line of identity is displayed for single-frequency bioimpedance analysis (SFBIA), multi-frequency bioimpedance analysis (MFBIA) and dual-energy X-ray absorptiometry (DXA) comparisons. Root mean square error (RMSE) and coefficient of determination (*R*^2^) are also presented. Results of muscle quality index (MQI_TOTAL_) are displayed for MFBIA *v*. DXA (Fig. 2(a)), SFBIA *v*. DXA (Fig. 2(c)) and SFBIA *v*. MFBIA (Fig. 2(e)). *Bland*–*Altman Analysis:* The relationship between the average of the MQI_TOAL_ estimates and a reference method (*x*-axis) and the difference in the estimate minus that of the reference method (*y*-axis) is displayed. The linear regression line indicates the degree of proportional bias. Horizontal dashed lines indicate the upper and lower limits of agreement (LOA), and the horizontal solid line indicates the constant error between methods. Linear regression equations and 95 % LOA values are also displayed. Results of MQI_TOTAL_ are displayed for MFBIA *v*. DXA (Fig. 2(b)), SFBIA *v*. DXA (Fig. 2(d)) and SFBIA *v*. MFBIA (Fig. 2(f)).

Correlations between MQI metrics ranged from 0·71 to 0·94 (Fig. 1). Strong, statistically significant correlations were observed for all ALM variables (0·84 < *R^2^* < 0·93; *P* < 0·001), with Lin’s concordance correlation coefficient values of 0·91–0·95 (Table 2). The slope and intercept of the Deming regression line did not differ from 1 and 0, respectively, for ALM_DXA_ *v*. ALM_SFBIA_ and MQI_DXA_ *v*. MQI_MFBIA_ but significantly differed for ALM_DXA_ *v*. ALM_MFBIA_, as well as MQI_DXA_ *v*. MQI_SFBIA_ and ALM and MQI comparisons for MFBIA *v*. SFBIA (Figs. 2 and 3). Statistical equivalence was demonstrated for DXA *v*. SFBIA (ALM_TOTAL_ and MQI_TOTAL_), but not for other comparisons. From Bland–Altman analysis, no proportional bias was observed for ALM_DXA_ *v*. ALM_SFBIA_ or MQI_DXA_ *v*. MQI_MFBIA_, but slight proportional bias (|slope| ≤ 0·14) was observed for other comparisons.

### Arm lean mass outcomes

**Fig. 4.**
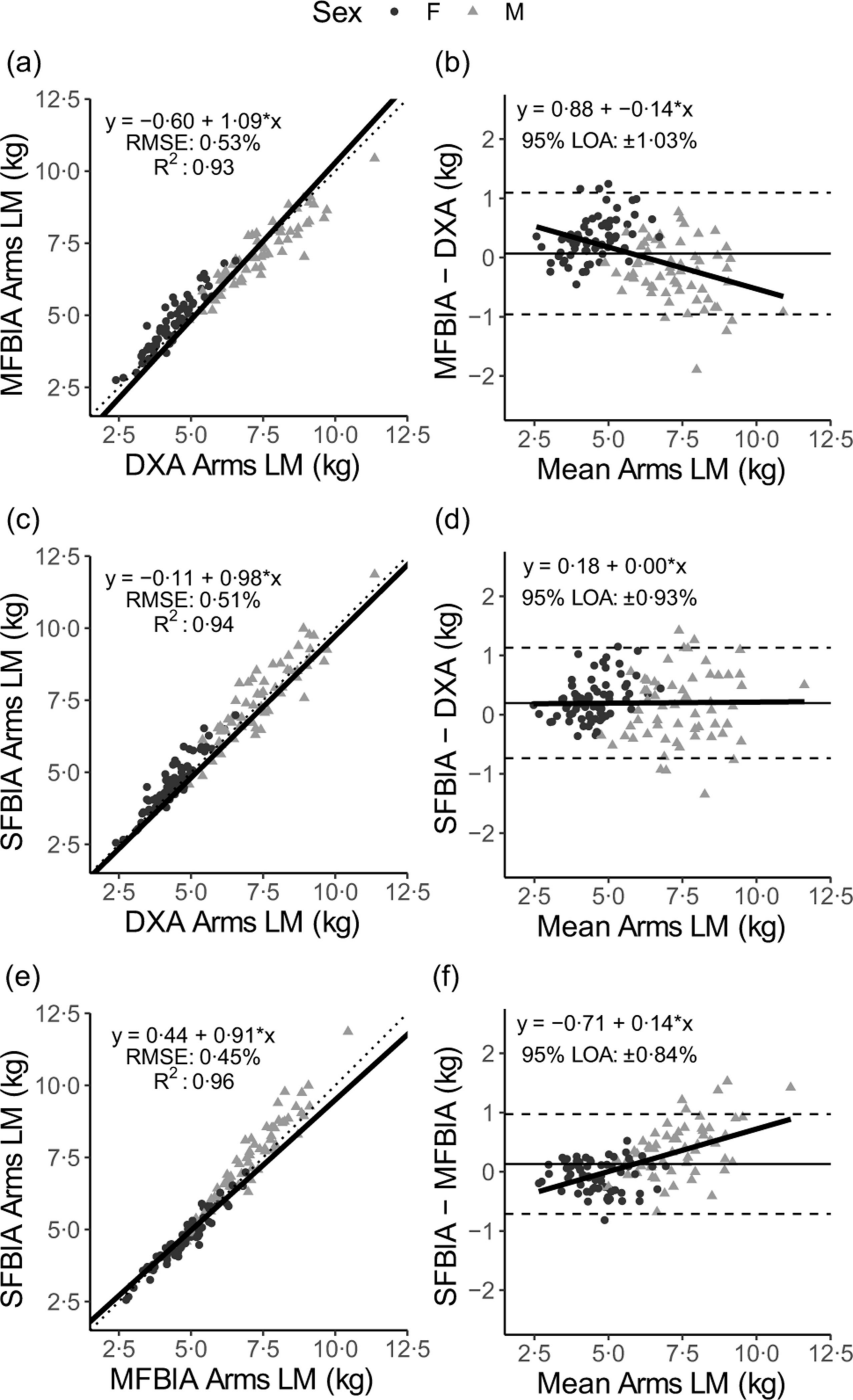
Comparison of body composition devices for estimating arms lean mass. *Line of Identity:* The ordinary least squares regression line as compared with the line of identity is displayed for single-frequency bioimpedance analysis (SFBIA), multi-frequency bioimpedance analysis (MFBIA) and dual-energy X-ray absorptiometry (DXA) comparisons. Root mean square error (RMSE) and coefficient of determination (*R*^2^) are also presented. Results of arms lean mass (LM) are displayed for MFBIA *v*. DXA (Fig. 2(a)), SFBIA *v*. DXA (Fig. 2(c)) and SFBIA *v*. MFBIA (Fig. 2(e)). *Bland*–*Altman Analysis:* The relationship between the average of the arms LM estimates and a reference method (*x*-axis) and the difference in the estimate minus that of the reference method (*y*-axis) is displayed. The linear regression line indicates the degree of proportional bias. Horizontal dashed lines indicate the upper and lower limits of agreement (LOA), and the horizontal solid line indicates the constant error between methods. Linear regression equations and 95 % LOA values are also displayed. Results of arms LM are displayed for MFBIA *v*. DXA (Fig. 2(b)), SFBIA *v*. DXA (Fig. 2(d)) and SFBIA *v*. MFBIA (Fig. 2(f)).

**Fig. 5.**
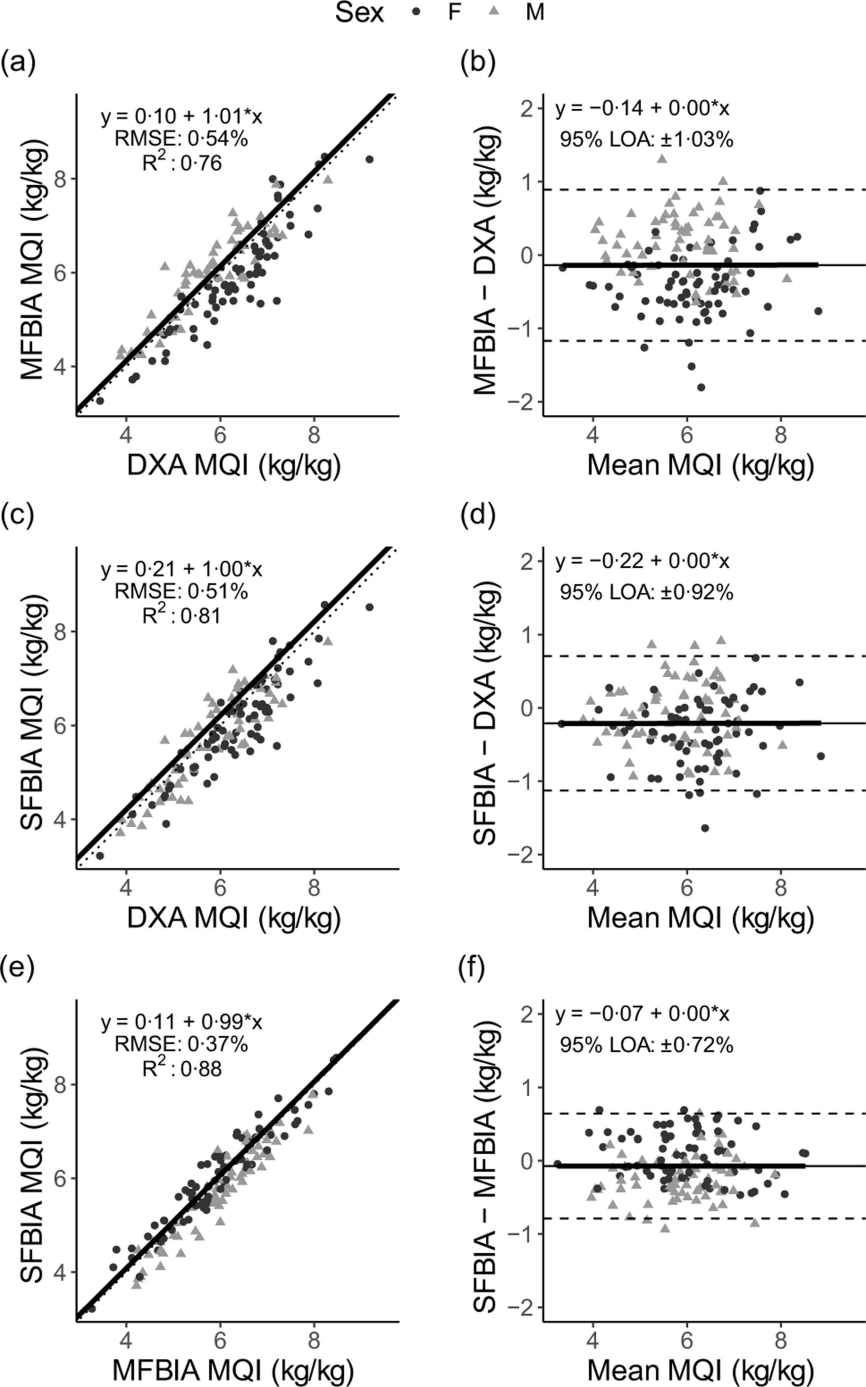
Comparison of body composition devices for measuring muscle quality index in arms (MQI_ARMS_). *Line of Identity:* The ordinary least squares regression line as compared with the line of identity is displayed for single-frequency bioimpedance analysis (SFBIA), multi-frequency bioimpedance analysis (MFBIA) and dual-energy X-ray absorptiometry (DXA) comparisons. Root mean square error (RMSE) and coefficient of determination (*R*^2^) are also presented. Results of arms muscle quality index (MQI_ARMS_) are displayed for MFBIA *v*. DXA (Fig. 2(a)), SFBIA *v*. DXA (Fig. 2(c)) and SFBIA *v*. MFBIA (Fig. 2(e)). *Bland*–*Altman Analysis:* The relationship between the average of the MQI_ARMS_ estimates and a reference method (*x*-axis) and the difference in the estimate minus that of the reference method (*y*-axis) is displayed. The linear regression line indicates the degree of proportional bias. Horizontal dashed lines indicate the upper and lower limits of agreement (LOA), and the horizontal solid line indicates the constant error between methods. Linear regression equations and 95 % LOA values are also displayed. Results of MQI_ARMS_ are displayed for MFBIA *v*. DXA (Fig. 2(b)), SFBIA *v*. DXA (Fig. 2(d)) and SFBIA *v*. MFBIA (Fig. 2(f)).

Strong, statistically significant correlations were observed for all variables (0·87 < *R^2^* < 0·98; *P* < 0·001), with Lin’s concordance correlation coefficient values of 0·86–0·97 (Table 2). The slope and intercept of the Deming regression line did not differ from 1 and 0, respectively, for ARMS_DXA_ *v*. ARMS_SFBIA_ or any MQI_ARMS_ but significantly differed for ARMS_DXA_ *v*. ARMS_MFBIA_ and ARMSMFBIA *v*. ARMS_SFBIA_ (Figs. 4 and 5). Statistical equivalence was demonstrated for ARMS_DXA_ *v*. ARMS_MFBIA_ and MFBIA *v*.SFBIA (MQI_ARMS_), but not other comparisons. From Bland–Altman analysis, no proportional bias was observed for ARMS_DXA_ *v*. ARMS_SFBIA_ or any MQI_ARMS_ but slight proportional bias (|slope| ≤ 0·14) was observed for other comparisons.

## Discussion

The purpose of this study was to compare SFBIA and MFBIA devices against DXA for ALM and MQI metrics in Hispanic adults. Results demonstrated that SFBIA and MFBIA had strong correlations with DXA for all ALM and MQI metrics. In addition, equivalence testing varied between methods (e.g. SFBIA *v*. DXA) when examining the different metrics (i.e. ALM_TOTAL_, ALM_ARMS_, MQI_TOTAL_, and MQI_ARMS_). Lastly, there was proportional bias, albeit slight, for multiple comparisons between the bioimpedance devices and DXA when evaluating ALM and MQI. Nonetheless, MQI_ARMS_ was the only metric that did not differ from the line of identity and had no proportional bias when comparing all the devices against each other. These findings could be an indicator that MQI_ARMS_, rather than MQI_TOTAL_, may be better to use when there are different body composition techniques being administered across multiple research and clinical settings. It is also possible that MQI_ARMS_ performed better due to the use of a measure of upper body strength with ALM_ARMS_. To support this postulation, future research may seek to evaluate MQI models that use lower body strength tests and ALM_TOTAL_ and ALM_LEGS_.

Comparisons between bioimpedance devices and DXA have shown mixed results when seeking to estimate body composition in the upper and lower extremities. For example, Esco *et al*.^(19)^ found MFBIA and DXA had excellent agreement when used to predict appendicular lean soft tissue (i.e. arms and legs) in collegiate female athletes. It is worth noting the lean soft tissue measures from Esco *et al*.^(19)^ excluded bone tissue. Contrarily, Brewer *et al*.^(30)^ found that MFBIA significantly underestimated ALM when compared against DXA in Division I college athletes. In addition, Nickerson^(15)^ found large mean differences between SFBIA and DXA when comparing arms, legs, and total ALM in physically active adults. However, the 95 % limits of agreement were small for all the comparisons, which suggest there may have been fixed bias of the SFBIA device^(15)^. Collectively, the current study findings demonstrate good overall agreement between SFBIA, MFBIA and DXA for ALM_TOTAL_ and ALM_ARMS_ in a Hispanic population.

The comparison of MQI between different body composition methods is limited. Nonetheless, a previous study found a strong association (*r* = 0·81; *P* < 0·001) between a field- and laboratory-based model using BMI and DXA, respectively^(31)^. Something worth highlighting is BMI and DXA utilise different metrics (kg/m^2^ and kg, respectively). Therefore, analysis in previous research was limited to correlations and not equivalence testing and Bland–Altman analysis^(31)^. Accordingly, the current study adds to previous literature by employing identical body composition metrics (i.e. ALM_TOTAL_ and ALM_ARMS_) across multiple devices (i.e. SFBIA, MFBIA, and DXA), which allows for a more comprehensive interpretation and rigorous statistical analysis. This brings forth a common issue in the literature which includes the use of different methods for measuring body composition and muscular strength components of MQI. For example, body composition can be measured with DXA, BIA, BMI, magnetic resonance imaging or computed tomography when calculating MQI. Moreover, muscular strength can be measured using grip strength, chair stand test, leg extensions, etc.^(1)^. Altogether, the lack of consensus on which methods to use when quantifying MQI makes comparing previous research extremely difficult.

The similar agreement between all three body composition methods when predicting MQI_ARMS_ is a talking point worth further discussion. For example, previous research from Nickerson^(15)^ revealed the agreement between SFBIA and DXA varies based on sex and segmental mass. Specifically, results demonstrated the error of SFBIA, when predicting segmental lean mass, was larger for males than females. One potential explanation of the increased error of SFBIA, when compared with DXA, was attributed to the larger segmental mass of males than females^(15)^. Accordingly, it is plausible the SFBIA and MFBIA devices in the current study have better agreement with DXA when used to predict MQI_ARMS_ than MQI_TOTAL_ since the former muscle quality metric has less segmental mass than the latter. The use of MQI_ARMS_ may also be more sensitive for detecting sex differences than MQI_TOTAL_. For instance, Lopes *et al*.^(32)^ found MQI was higher in females than males when using dominant HGS and the corresponding arm’s ALM^(32)^. Contrarily, there were no differences between males and females when comparing MQI_TOTAL_ (i.e. combined HGS and ALM_TOTAL_)^(32)^. The current study is the first ever to demonstrate similarity between MQI_ARMS_ and differences amongst MQI_TOTAL_ when comparing multiple body composition methods with similar body composition metrics (i.e. ALM). These findings highlight the need to further explore MQI models when using various body composition tools, muscular strength methods (e.g. handgrip, chair stands, leg extension) and ALM measures (e.g. arms, legs, combined).

Although the current study has many strengths, it is not without limitations. First, it is worth mentioning the present study utilised young- and middle-aged adults. As a result, it is unknown whether the current study findings can be generalised to older adults. MQI is commonly evaluated in older adults due to loss of muscular strength and ALM, which is associated with ageing. Nonetheless, MQI is important to evaluate across various age spectrums, including young- and middle-aged adults, particularly those interested in training interventions designed to improve physical functioning. Second, the current study sample consisted of Hispanic adults. Consequently, it is unknown whether the present study findings can be generalised to non-Hispanic populations. Nonetheless, most of the research, regarding MQI, has been centred on non-Hispanic populations. Thus, the present study filled a gap in the literature by evaluating a population that has been underrepresented in body composition research. Altogether, the present study results should only be generalised to Hispanic adults 18 – 55 years of age. Third, it should be noted that current study results only apply to the SFBIA and MFBIA devices utilised in the present study. Numerous BIA devices are commercially available for use. Therefore, assuming results apply to all SFBIA and MFBIA should be avoided until further research can be conducted utilising devices not included in the present study. Nonetheless, the present study uniquely showed that SFBIA and MFBIA have a similar agreement with DXA when used to predict ALM and MQI. The ability of MFBIA to utilise low and high frequencies is often assumed to result in better accuracy than simpler SFBIA technology, which uses a single low-frequency electrical current. However, our results demonstrate MFBIA does not result in better agreement than SFBIA. Thus, both devices yielded similar outcomes and are very promising for use when seeking to compute MQI. Lastly, the current study did not record the dominant hand of participants during testing. It is possible there are differences between dominant and non-dominant HGS. Therefore, the average HGS (left hand + right hand)/2 was used to compute MQI models in the current study. This approach likely helped minimise differences that may have existed between the dominant and non-dominant hand.

## Conclusion

Comparisons of BIA *v*. DXA for measuring MQI_TOTAL_ and MQI_ARMS_ have yet to be explored. Additionally, it was previously unknown whether various BIA devices (i.e. SFBIA and MFBIA) could be used interchangeably for measuring MQI, when compared with DXA. The current study uniquely showed that SFBIA and MFBIA have better agreement with DXA when used to compute MQI_ARMS_ than MQI_TOTAL_. These results have significant clinical implications when seeking to compute MQI with different body composition methods (i.e. DXA, MFBIA, and SFBIA). For example, MQI_ARMS_ is advised for research facilities and multi-site studies that comprise of different body composition methods. Furthermore, MQI_ARMS_ may be better to assess than MQI_TOTAL_ when patients visit numerous healthcare locations that utilise varying BIA models for analysis of ALM. Future steps include the following: (1). Evaluating BIA devices beyond the models examined in the present study; (2). Comparison of MQI across various races/ethnicities; (3). Steps toward a consensus on how to standardise the measurement of MQI; and (4). Longitudinal studies evaluating the associations between MQI and health-related outcomes in clinical populations undergoing prevention and treatment interventions (e.g. obesity, sarcopenia and cancer).

## Figures and Tables

**Table 1.** Subject characteristics mean and standard deviation (SD)

	All (*n* 131)		F (*n* 71)		M (*n* 60)	
			
	Mean	SD	Mean	SD	Mean	SD

Height (cm)	166·5	8·7	160·7	5·8	173·4	6·2
Weight (kg)	78·1	17·8	72·0	16·4	85·4	16·7
BMI (kg/m^2^)	28·1	5·8	27·9	6·3	28·3	5·1
Age (year)	29·1	11·3	29·9	11·2	28·2	11·5
Average handgrip strength (kg)	33·9	9·2	27·0	4·8	42·1	6·0

**Table 2.** Comparisons between SFBIA, MFBIA and DXA for ALM and MQI

		Variable 1		Variable 2						TOST Interval		
					
Variable 1	Variable 2	Mean	SD	Mean	SD	CE	CE SD	SEE	CCC	LL	UL	Equivalence

ALM_DXA_ (kg)	ALM_MFBIA_	21·49	5·43	20·98	4·75	−0·51	1·49	1·23	0·95	−0·73	−0·30	N
ALM_DXA_ (kg)	ALM_SFBIA_	21·49	5·43	21·65	5·31	0·16	1·62	1·58	0·95	−0·07	0·40	Y
ALM_MFBIA_ (kg)	ALM_SFBIA_	20·98	4·75	21·65	5·31	0·67	1·50	1·46	0·95	0·46	0·89	N
MQI_DXA-ALM_ (kg/kg)	MQI_MFBIA-ALM_	1·59	0·26	1·62	0·26	0·03	0·11	0·10	0·91	0·01	0·04	N
MQI_DXA-ALM_ (kg/kg)	MQI_SFBIA-ALM_	1·59	0·26	1·58	0·29	−0·01	0·11	0·11	0·92	−0·03	0·01	Y
MQI_MFBIA-ALM_ (kg/kg)	MQI_SFBIA-ALM_	1·62	0·26	1·58	0·29	−0·04	0·10	0·10	0·93	−0·05	−0·02	N
Arms LM_DXA_ (kg)	Arms LM_MFBIA_	5·74	1·86	5·81	1·62	0·07	0·52	0·43	0·95	−0·01	0·14	Y
Arms LM_DXA_ (kg)	Arms LM_SFBIA_	5·74	1·86	5·94	1·87	0·20	0·48	0·47	0·96	0·13	0·27	N
Arms LM_MFBIA_ (kg)	Arms LM_SFBIA_	5·81	1·62	5·94	1·87	0·13	0·43	0·38	0·97	0·07	0·19	N
MQI_DXA-ARMS_ (kg/kg)	MQI_MFBIA-ARMS_	6·08	1·04	5·94	1·04	−0·14	0·53	0·51	0·86	−0·21	−0·06	N
MQI_DXA-ARMS_ (kg/kg)	MQI_SFBIA-ARMS_	6·08	1·04	5·87	1·04	−0·21	0·47	0·46	0·88	−0·28	−0·14	N
MQI_MFBIA-ARMS_ (kg/kg)	MQI_SFBIA-ARMS_	5·94	1·04	5·87	1·04	−0·07	0·37	0·36	0·94	−0·13	−0·02	Y

TOST: two one-sided *t* tests; CE: constant error; SEE: standard error of the estimate; CCC: Lin’s concordance correlation coefficient; LL: lower limit; UL: upper limit; ALM = appendicular lean mass; MQI = muscle quality index; LM = lean mass; DXA = dual-energy X-ray absorptiometry; SFBIA = single-frequency bioimpedance analysis; MFBIA = multi-frequency bioimpedance analysis.

## Abbreviations:

ALM: appendicular lean mass
BIA: bioimpedance analysis
DXA: dual-energy X-ray absorptiometry
HGS: handgrip strength
MQI: muscle quality index
MFBIA: multi-frequency bioimpedance analysis
SFBIA: single-frequency bioimpedance analysis
